# Immune profiling of the bone marrow microenvironment in patients with high-risk localized prostate cancer

**DOI:** 10.18632/oncotarget.27817

**Published:** 2020-11-17

**Authors:** Erika Heninger, Nan Sethakorn, David Kosoff, Peiman Hematti, Morgan D. Kuczler, Kenneth J. Pienta, Joshua M. Lang

**Affiliations:** ^1^University of Wisconsin Carbone Cancer Center, Madison, WI, USA; ^2^Department of Oncology, University of Wisconsin, Madison, WI, USA; ^3^Department of Medicine, University of Wisconsin, Madison, WI, USA; ^4^Department of Oncology, Sidney Kimmel Comprehensive Cancer Center, The Johns Hopkins School of Medicine, Baltimore, MD, USA; ^5^Department of Urology, The James Buchanan Brady Urological Institute, Baltimore, MD, USA; ^6^Department of Pharmacology and Molecular Sciences, The Johns Hopkins School of Medicine, Baltimore, MD, USA

**Keywords:** prostate cancer, bone marrow, tumor microenvironment, immune profiling, immune landscape

## Abstract

Bone marrow (BM) is a primary metastatic site in prostate cancer (PC) and bone invasion is considered incurable. T cell-mediated immune surveillance is essential in controlling both tumorigenesis and initiation of metastases. Beside tropism, dissemination of PC cells to the BM may be facilitated by defects in BM immune homeostasis predisposing this niche to colonization.

To evaluate the BM immune microenvironment in locally advanced, non-metastatic PC, we performed flow cytometry analysis of myeloid and lymphoid subsets in BM aspirates and peripheral blood collected during prostatectomy. Healthy BM aspirates served to establish a reference range for comparison.

We found alterations in BM immune composition of PC patients, including an increased CD4/CD8 ratio, enrichment of CD4^+^ T cells, increased CD56^+^CD3^+^ NKT and CD56^+^CD3^-^ NK yields compared to healthy controls. The lymphoid phenotype remained comparable regarding T cell activation and chemokine receptor-based polarization patterns. Additionally, we found increased B7H3 expression in the myeloid monocyte/macrophage subset and decreased DC infiltration in BM of PC patients.

These findings suggest that alterations in the immune milieu may limit immune surveillance that compromise the ability of the BM microenvironment to prevent tumor dissemination, and predispose development of bone metastases in a subset of patients with localized PC.

## INTRODUCTION

The immune system provides critical protection against tumor progression and dissemination and can induce dramatic, even complete tumor regression in advanced stages of disease. Augmenting anti-tumor immune responses has become a successful cancer treatment strategy that has driven significant improvements in progression-free and overall survival for patients with a wide range of malignancies [1–5]. Unfortunately, immune targeting therapies have had limited success in the treatment of prostate cancer (PC) so far and the varied pathways by which prostate tumor cells are able to subvert immune responses continue to be under active investigation [6–11].

PC immune subversion within the bone marrow (BM) is an area of particular interest, which may have important implications for PC progression and high clinical relevance for patients. The BM is a crucial hematopoietic organ and a source of anti-tumor immune cells. Tumor reactive T cells in the BM have been shown to induce dramatic tumor regressions in an array of cancers and these T cells have even been found to be more potent than tumor reactive T cells in the peripheral circulation [12–14]. Yet, despite these potent anti-tumor properties, PC cells are able to disseminate to the BM with great frequency. The BM is the most common site of PC metastases and progression of these metastatic foci are a primary cause of morbidity and death for patients [15]. The establishment and progression of PC metastases within this potentially hostile environment suggests that PC induces alterations in BM immune homeostasis, which permits survival of metastatic foci. However, such alterations in the BM immune landscape, particularly in men with early state, localized disease, have not yet been clearly defined.

In this study, we aimed to identify alterations within the BM immune microenvironment that could promote the establishment and progression of BM metastases in men with clinically localized PC. We developed multiparameter flow cytometry assays to define the pre-metastatic immune profile in blood and BM samples from patients with localized, high-risk PC undergoing radical prostatectomy. In addition to activation and basic differentiation features of the lymphoid and myeloid compartments, we assessed a variety of chemokine-receptor profiles and expression of positive and negative co-stimulatory markers to gain more granularity of polarization status of both lymphoid and myeloid cells beyond composition. To our knowledge, this study provides the most comprehensive analysis of the BM immune microenvironment in patients with localized PC.

## RESULTS

In order to evaluate for alterations in the BM immune microenvironment of men with primary, localized PC, we developed two highly polychromatic flow cytometry assays to analyze and characterize the lymphoid and myeloid immune infiltrate within the BM. We included markers for several main lymphoid and myeloid immune subsets as well as markers for polarization and activation (markers summarized in Supplementary Table 1). We then employed these assays to analyze the BM aspirates of men with localized PC as well as healthy controls. Our analysis included 27 BM aspirates that were collected at the time of radical prostatectomy from patients with primary, localized PC and 10 healthy BM donor aspirates. Additionally, in a partial cohort of 14 PC patients and 6 healthy donors the immune analysis was expanded to evaluate cell activation and polarization patterns of BM infiltrating immune subsets. For the men in the PC cohort, we also obtained matched peripheral blood (PB) samples to analyze along with their BM aspirates. Assay and instrument standardization protocols were established using Mid-Range Ultra-Comp Rainbow beads. Analysis was performed using the FlowJo software. Debris, dead cells and aggregates were excluded from analysis to reduce autofluorescence. After we established our protocol and gating strategies, we analyzed the lymphoid and myeloid composition of PC BM aspirates and compared these findings to the panel of healthy donors.

### Lymphoid immune subsets

**Figure 1:**
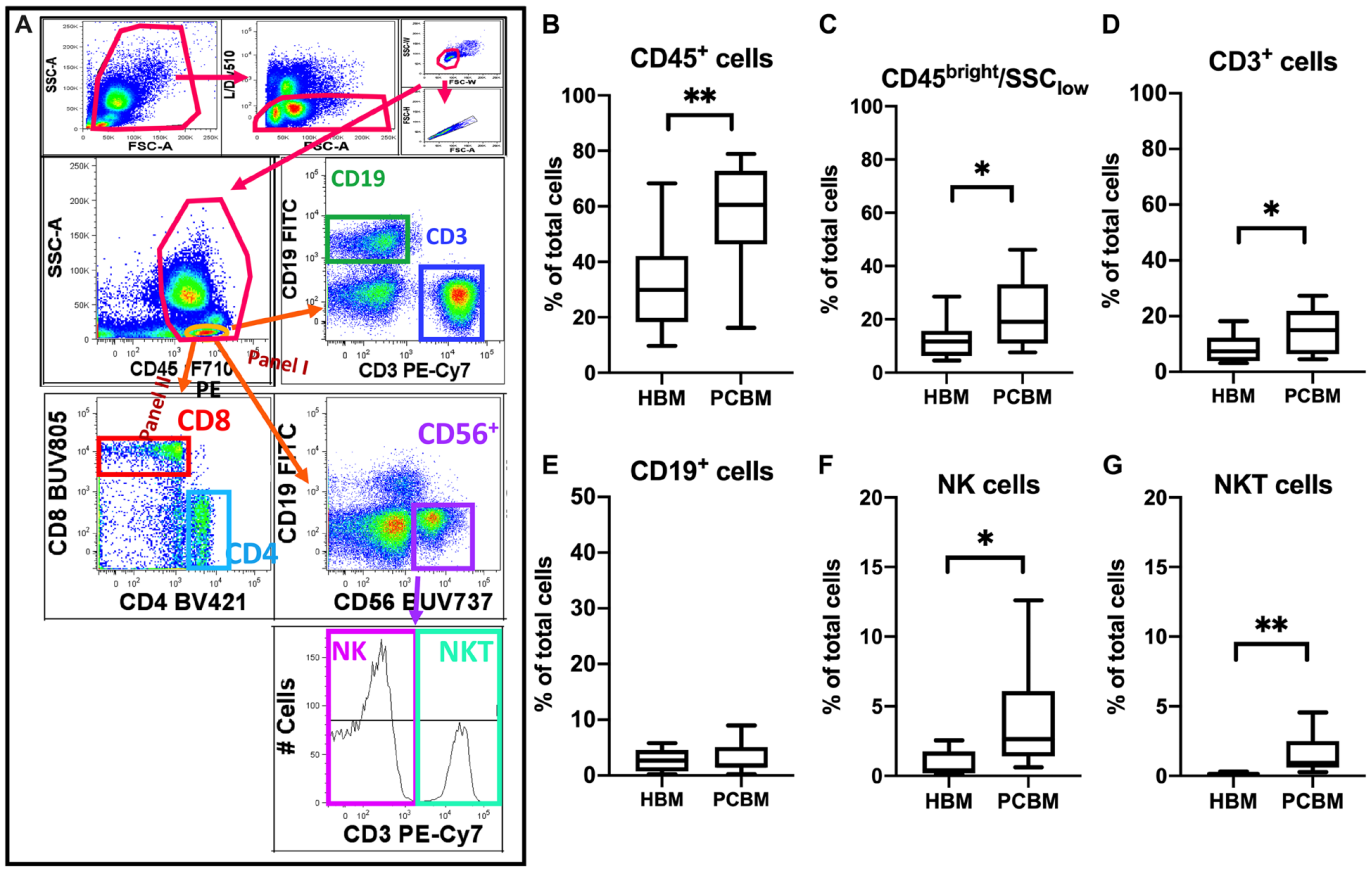
Flow cytometry analysis of lymphoid cellular subsets in BM aspirates. Flow cytometry analysis in (**A**) represents the gating strategy utilized to analyze lymphoid immune subsets. The top row shows basic quality control gating to reduce autofluorescence by excluding debris, dead cells and aggregates using FSC, SSC Area (A), Height (H) and Width (W) and live/dead staining. Total CD45^+^ cells were then gated and lymphoid cells were conventionally defined as the CD45^bright^/SSC_low_ subset (circled in orange in second row, left dot plot) followed by extraction of the CD3^+^ and CD19^+^ events in Panel I. NK and NKT cells were sub-gated within the CD56^+^ lymphoid subset as CD3^-^ and CD3^+^, respectively. Box plots represent the frequency of (**B**) total CD45^+^ events (**C**) CD45^bright^/SSC_low_ lymphoid cells (CD45^+^ Ly) (**D**) CD3^+^/CD45^bright^/SSC_low_ (**E**) CD19^+^/CD45^bright^/SSC_low_ (**F**) CD56^+^/CD3^-^/CD45^bright^/SSC_low_ NK cells and (**G**) CD56^+^/CD3^+^/CD45^bright^/SSC_low_ NKT cells within the total single/live/cells infiltrate. Box plots present data distribution with median line HBM *n* = 10, PCBM *n* = 14 (B–E); HBM *n* = 6, PCBM *n* = 11 (F–G). ^*^ *p* < 0.05; ^**^ *p* < 0.01.

We first evaluated the basic lymphoid composition of BM aspirates (Figure 1). The gating strategy is represented in Figure 1A. Compared with healthy donors, we observed that the frequency of CD45^+^ cells was significantly higher in the PC BM aspirates (31.56% vs 58.81%, *p* = 0.0016) and that within this CD45 population, there was an enrichment of total CD45^+^ lymphocytes (CD45^bright^/SSC_low_) (12.65% vs 22.04%, HBM vs PC, respectively, *p* = 0.0368). On analysis of the lymphocyte subset, we found an increase of CD3^+^ T cells (CD3^+^/CD45^bright^/SSC_low_) (8.503% vs 14.09%, *p* = 0.0469) while frequency of CD19^+^ B cells (CD19^+^/CD45^bright^/SSC_low_) remained comparable between the two groups. The yield of NK cells (CD56^+^/CD3^-^/CD45^bright^/SSC_low_) was significantly higher in PC marrow (0.8727% vs 3.852%, *p* = 0.0224) and there was a 10-fold enrichment of BM infiltrating NKT cells (CD56^+^/CD3^+^/CD45^bright^/SSC_low_) in PC BM compared to healthy donors (0.1502% vs 1.558%, *p* = 0.0077). The increase in NKT frequencies was also significantly higher in PC BM vs HBM when normalized to the CD45^+^ infiltrate (0.4957% in HBM vs 3.109% in PCBM, *p* = 0.0052, data not shown).

**Figure 2:**
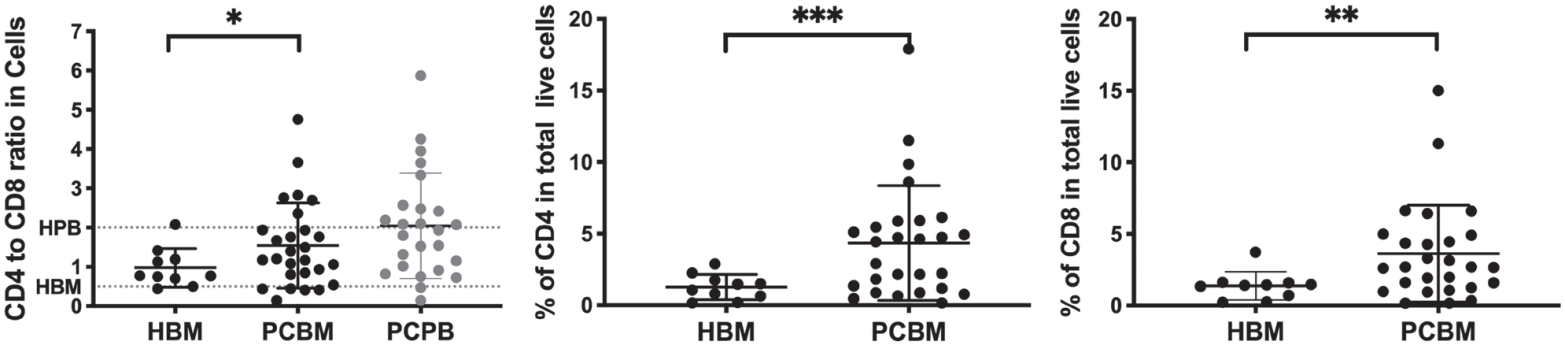
Flow cytometry analysis of the CD4 to CD8 ratio. Dot plot on left shows the CD4 to CD8 ratio in healthy BM aspirates (HBM in black) PC patient BM aspirates (PCBM in black) and PC patient peripheral blood (PCPB in grey). Dotted lines represent previously reported expected reference values for healthy peripheral blood (HPB) and healthy bone marrow (HBM). The dot plots in the middle and right represent frequencies of CD4^+^ or CD8^+^ cells in total live cell infiltrate. Data is presented as individual data points with Mean ± SD. HBM *n* = 10, PCBM *n* = 27, PCPB *n* = 24. ^*^ *p* < 0.05; ^**^ *p* < 0.01.

To assess the lymphoid compartment with more granularity, we evaluated the ratio of CD4^+^ T cells and CD8^+^ T cells (Figure 2). We observed a significant increase of the CD4/CD8 ratio in total live cells in PC BM samples compared to healthy aspirates (mean of 0.97 vs 1.54, HBM vs PCBM, respectively, *p* = 0.0363). The CD4 to CD8 ratio was similarly higher in PC BM when normalized to the CD45^+^ infiltrate (mean of 0.97 vs 1.51, HBM vs PCBM, respectively, *p* = 0.0441) or to the CD45^bright^/SSC_low_ lymphocyte subset (mean of 0.97 vs 1.53, HBM vs PCBM, respectively, *p* = 0.0387) (Supplementary Figure 1A and 1B, respectively). We have analyzed the CD4/CD8 ratio of a total of 24 PB samples from the enrolling PC patients (22 out of 24 were matched to BM aspirates), which resulted an average of 2.04, matching the expected healthy reference [16–18]. To identify the factor behind the skewed CD4 to CD8 ratio in BM, we looked at the frequency of both CD4^+^ T cells and CD8^+^ T cells in the aspirates. We detected a significant increase in both CD4 and CD8 frequencies of the total live cell content when compared the PC cohort to healthy donor samples (4.3% vs 1.3%, *p* = 0.0007 and 3.6% vs 1.4%, *p* = 0.0037, PC vs HBM, respectively), however, the fold increase was higher in the CD4 subset than in CD8 (3.4 vs 2.6-fold, respectively) suggesting a robust CD4 infiltration in PC patients accounting for the skewed CD4 to CD8 ratio.

**Figure 3:**
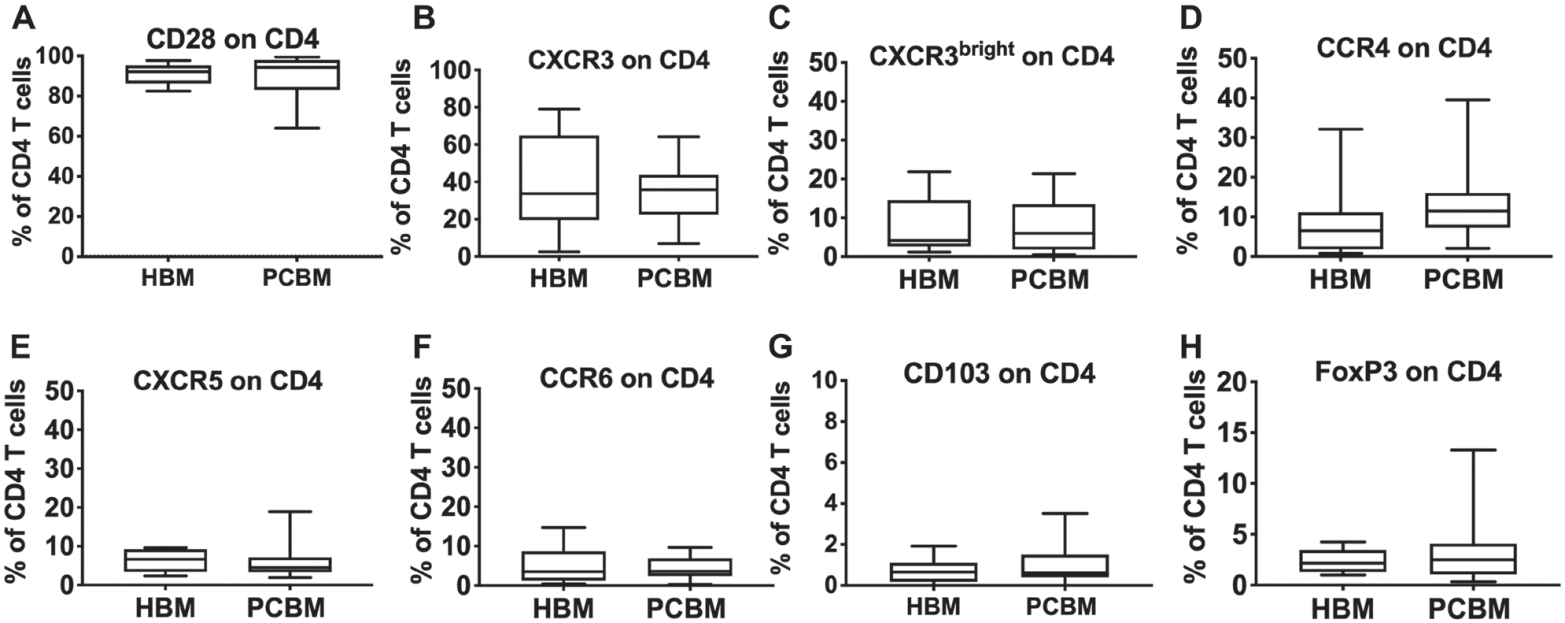
Flow cytometry analysis of CD4 infiltrates. Graphs represent frequency of CD28 (**A**), CXCR3 (**B**), CXCR3^bright^ (**C**), CCR4 (**D**), CXCR5 (**E**), CCR6 (**F**), CD103 (**G**) or FoxP3 (**H**) positive events within the CD4^+^ gate. HBM *n* = 10, PCBM *n* = 14 (A–G), PCBM *n* = 27 (H). Box plots represent data distribution with median line, Min to Max.

**Figure 4:**
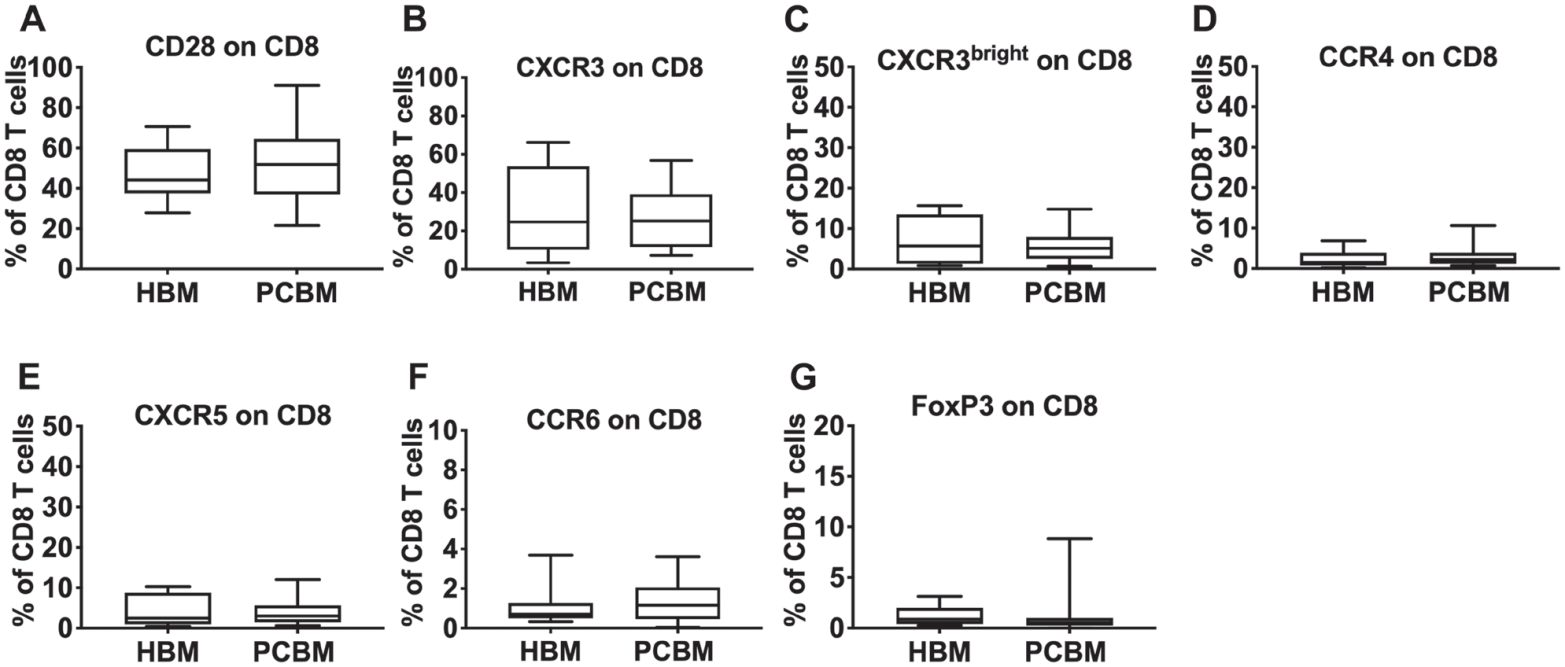
Flow cytometry analysis of CD8 infiltrates. Graphs represent frequency of CD28 (**A**), CXCR3 (**B**), CXCR3^bright^ (**C**), CCR4 (**D**), CXCR5 (**E**), CCR6 (**F**) or FoxP3 (**G**) positive events within the CD8^+^ gate. HBM *n* = 10, PCBM *n* = 14 for (A–F), PCBM *n* = 27 (G). Box plots with whiskers represent data distribution with median line, Min to Max.

Next, we looked at the phenotypic characteristics of infiltrating CD4^+^ (Figure 3) and CD8^+^ T cells (Figure 4). We analyzed T cell activation and found that expression levels of CD28 in both the CD4^+^ and CD8^+^ subsets were comparable with the healthy cohort (Figures 3A and 4A, respectively). We then analyzed the expression of various chemokine receptors (CXCR3, CCR4, CXCR5, CCR6) on both CD4 (Figure 3) and CD8 T cells (Figure 4) that are characteristically expressed by distinct functional subsets including Th1/Tc1, Th2/Tc2, Tfh/Tfc, Th17/Tc17, respectively [19]. CXCR3 is considered an inflammatory chemokine receptor, that is rapidly induced on activated T cells and remains preferentially and highly expressed on Th1-type CD4 cells and on cytolytic CD8 T cells [20]. We have measured the frequency of both total CXCR3^+^ cells (Figures 3B and 4B) and the CXCR3^bright^ subset (Figures 3C and 4C) within the CD4 and CD8 cells. Gating strategies for the T cell analysis are shown in Supplementary (Supplementary Figure 2A).

Chemokine expression patterns for CXCR3, CXCR3^bright^, CCR4, CXCR5, CCR6, (Figures 3 and 4B–4F respectively) were comparable between the PC patient group and healthy aspirates. Similarly, we found no differences in the frequency of CD103^+^/CD4^+^ (Figure 3G) cells between the two groups.

We assessed the intracellular expression of the FoxP3 regulatory T cell marker in both the CD4^+^ and CD8^+^ T cell subsets (Figures 3H and 4G, respectively) and found no evidence of regulatory T cell enrichment in the bone marrow aspirates of PC patients. We also calculated the CD8 to T_reg_ (CD8^+^ to CD4^+^FoxP3^+^) and Th17 to T_reg_ ratios (CD4^+^CCR6^+^ to CD4^+^FoxP3^+^) and observed no differences (data not shown).

### Myeloid immune subsets

**Figure 5:**
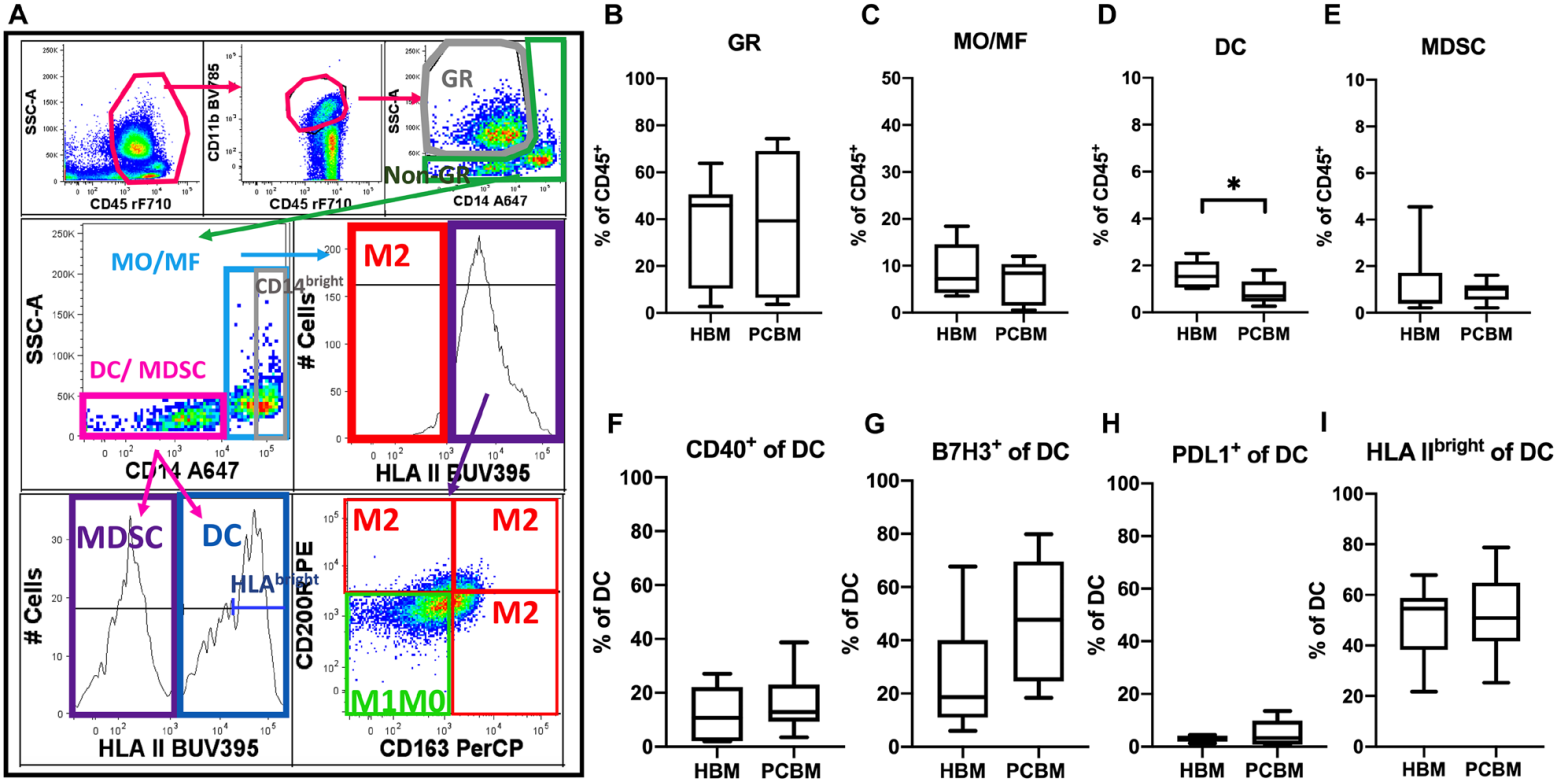
Flow cytometry analysis of myeloid cellular subsets in BM aspirates. (**A**) Represents the myeloid gating strategy. The CD45^+^ subset was extracted after exclusion of debris, dead cells and aggregates, followed by gating on the CD11b pan-marker for myeloid cells. Within the CD11b^+^ subset, we have outlined granulocytes (GR) as pictured on the dot plot on top far right (CD14_low_/SSC_high_). Then, we excluded GR cells with a Boolean invert gating strategy (non-GR) and projected these events on the CD14 expression spectrum (middle row, left). The CD14^-^ cells (including DC and MDSC) were then sub-gated for HLA II expression (bottom row, left). HLA II^+^ events were defined as a subset that enriched in CD11b^+^ dendritic cells (DC). The HLA II^-^ events contain myeloid-derived stem cells (MDSC) beside other progenitors not characterized further in this study. Monocytes and macrophages (MO/MF) were then gated as the CD14^+^/non-GR component (middle row, left). M1M0 and M2-like MO/MF were enriched by a Boolean gating strategy as follows. M1M0-like cells were defined as HLAII^+^ AND NOT expressing CD200R OR CD163^bright^ (lower left quadrant in dot plot in bottom row, left; M1M0-like = green box). MO/MF cells that were either HLA II^-^ (histogram in middle row; red box) OR HLAII^+^/CD163^bright^/CD200R^-^ OR HLAII^+^/CD163^low^/CD200R^+^ OR HLAII^+^/ CD163^bright^/CD200R^+^ (bottom row right) were rendered in a Boolean gate that pooled and enriched M2-like cells. Box plots in the first row represent frequencies of enriched (**B**) granulocytes (CD11b^+^/CD14_low_/SSC_high_), HBM *n* = 10, PCBM *n* = 14; (**C**) monocyte/macrophages (MO/MF; CD11b^+^/nonGR/CD14_high_); (**D**) CD11b^+^ dendritic cells (DC; CD11b^+^/nonGR/CD14_low_/HLA II^+^); and (**E**) myeloid-derived stem cells (MDSC; CD11b^+^/nonGR/CD14_low_/HLA II^-^). Graphs in the second row represent analysis of CD11b^+^ dendritic cells in BM aspirates. Data represent percent of CD40^+^, B7H3^+^, PDL1^+^ and HLA II^bright^ events within the DC subset (**F**–**I**, respectively). Box plots with whiskers represent data distribution with median line, min to max.; HBM *n* = 6, PCBM *n* = 11 (C–I). ^*^ *p* < 0.05.

To identify and define distinct myeloid subsets, we employed both specific surface markers and conventional indirect methods [21] using side scatter (SSC) and Boolean gating strategies depicted in Figure 5A. First, we gated CD45^+^ cells that co-expressed the CD11b pan-marker, then gated on the CD14_low_ events that also scattered high on SSC to separate granulocytes (GR) (CD11b^+^/CD14_low_/SSC_high_) [21]. Next, we gated on the CD11b^+^ cells and excluded the GR-like events (CD11b^+^/CD14_low_/SSC_high_) (gating strategy shown in Figure 5A) [21] to further enrich for other myeloid subsets including monocytes, macrophages, dendritic cells, myeloid-derived suppressor cells (MDSCs). To enrich the monocyte/macrophage (MO/MF) fraction, we gated on CD14^+^ events. Within the CD14^-^ gate, we defined CD11b^+^ dendritic cells (DC) as the HLA II^+^ fraction and MDSCs as the HLA II^-^ events.

We established the frequency of the myeloid subsets within the total CD45^+^ infiltrate including granulocytes (GR = CD11b^+^/CD14_low_/SSC_high;_ non-GR = Boolean inverted GR gate), monocytes/macrophages (MO/MF = nonGR/CD11b^+^/CD14_high_), CD11b^+^ dendritic cells (DC = nonGR/CD11b^+^/CD14_low_ HLA II^+^) and MDSCs (nonGR/CD11b^+^/CD14_low_ HLA II^-^) (Figure 5B–5E, respectively). While granulocyte, monocyte/macrophage and MDSC yields were all comparable between the PC and healthy cohort, we detected a significant decrease in the frequency of the CD11b^+^ DC subset in the PC group versus the healthy cohort (1.622% vs 0.8671%, *p* = 0.0347).

**Figure 6:**
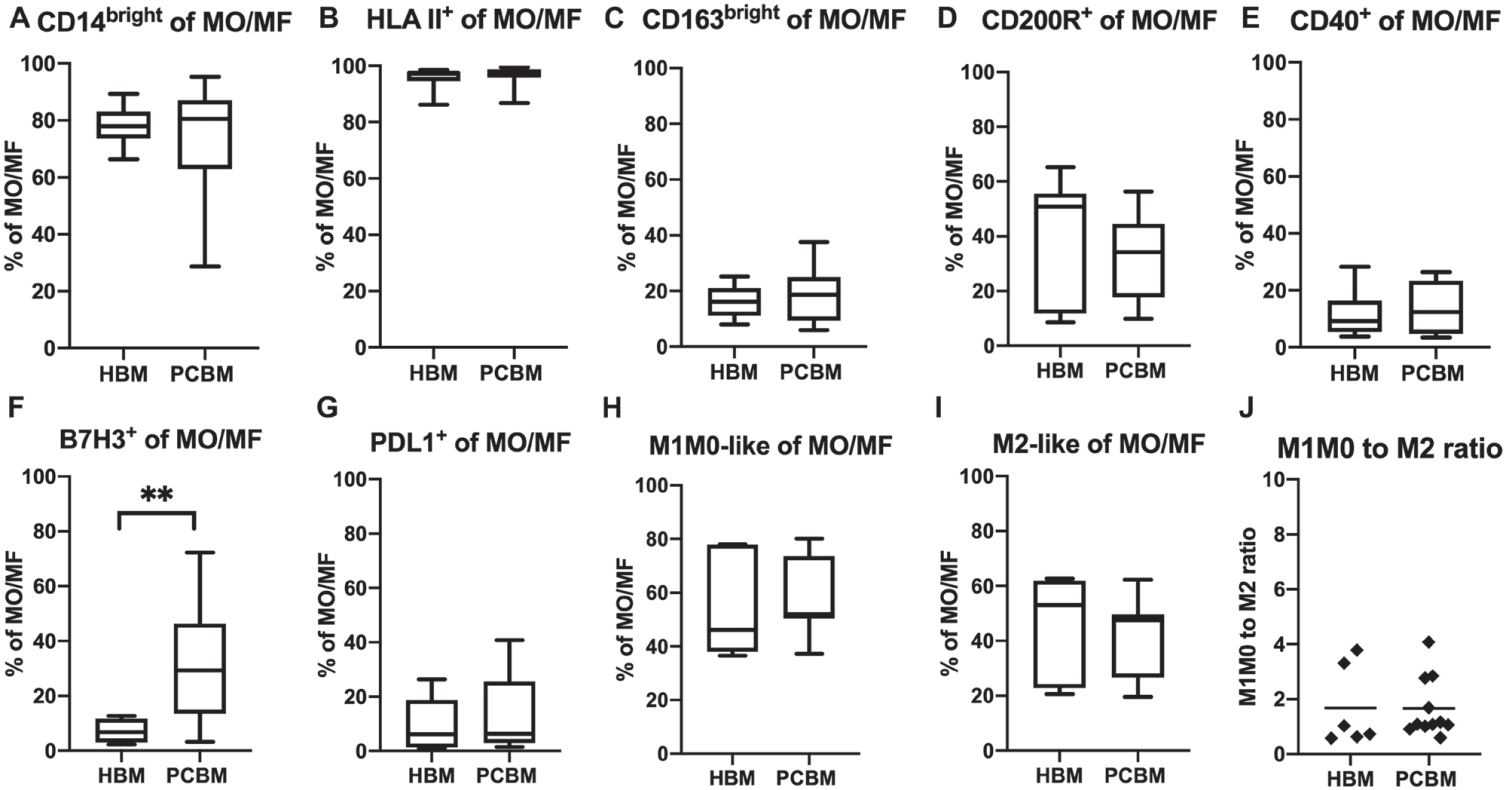
Monocyte/macrophage polarization in BM aspirates. Data represents frequency of cells expressing (**A**) CD14^bright^, (**B**) HLA-II^+^, (**C**) CD163^bright^, (**D**) CD200R, (**E**) CD40, (**F**) B7H3, (**G**) PDL1 protein within the total monocyte/macrophage subset. Box plots in (**H**–**J**) represent M1/M2 polarization in BM aspirates. Data shows frequency of enriched M1M0-like (H) or M2-like (I) monocyte/macrophages within the total MO/MF subset. All box plots with whiskers represent data distribution with median line, Min to Max. The dot plot (J) represents the M1M0 to M2 ratio within each sample with Mean. HBM *n* = 6, PCBM *n* = 11. ^*^ *p* < 0.05, ^**^ *p* < 0.01.

Next, we assessed polarization patterns within the myeloid immune microenvironment and compared that between a cohort of 11 PC patients and 6 healthy donors. We first analyzed the phenotype of the CD11b^+^ DC subset and found comparable expression levels of CD40 (Figure 5F). Next, we analyzed the expression of two negative checkpoint molecules from the B7-H family on DCs, B7H3 and PDL1 (aka B7H1). While we observed some increase of B7H3 expression in the PC cohort, that remained only a tendency (Figure 5G). PDL1 expression was comparable between the two cohorts (Figure 5H). The expression of HLA II (DR, DP, DQ) was also similar between the groups (Figure 5I). To further assess the profile of the MO/MF subset in BM aspirates (Figure 6), we analyzed the expression levels of several activation and polarization markers including CD14^bright^, HLA II, CD163^bright^, CD200R (Figure 6A–6D, respectively) and found them all comparable between the two cohorts. In addition, we measured expression of co-stimulatory molecules including positive co-stimulatory CD40 (Figure 6E), and negative check-point molecules B7H3 and PDL1 (Figure 6F and 6G, respectively). We have found a robust increase in the frequency of B7H3^+^ MO/MF cells between the healthy donor vs PC patient aspirates (7.2% vs 32.3%, *p* = 0.0052) while the expression of both PDL1 and CD40 were comparable on MO/MF cells between the groups. Next, we aimed to analyze macrophage polarization patterns in the BM aspirates. Alternative macrophage polarization has been associated with tumor-promoting microenvironments. CD163 is a hemoglobin scavenger receptor and is a macrophage-exclusive protein [22]. CD200R is an immune-inhibitory protein that is expressed more broadly by myeloid cells and T cells [23, 24]. The upregulation of both of these proteins have been associated with alternative M2-like macrophage activation. We established a gating strategy to enrich for M2-like and M1M0-like subsets within the MO/MF population (Figure 5A). To enrich the M2-like MO/MF cells, we pooled events that were either HLAII^-^ OR HLAII^+^/CD163^bright^/CD200R^-^ OR HLAII^+^/CD200R^+^/CD163^low^ OR HLAII^+^/CD200R^+^/CD163^bright^ in a Boolean gate. The exclusion HLAII^+^/CD163^low^/CD200R^-^ MO/MF subset enriched M1-like and M0 unpolarized cells, defined as ‘M1M0-like’ in our gating strategy. In Figure 6H and 6I, we show the frequencies of the enriched M1M0-like or M2-like MO/MF subsets, respectively. The ratio of these two subpopulations has been calculated and shown in Figure 6J. We did not find evidence of an M1/M2 polarization shift in BM in the PC group.

### Clinical features and immunologic correlations

Finally, we obtained clinical outcome data for our patient cohort, summarized in Table 1. Median age of patients was 65 year (49–76). 3.23% of the cases (1 out of 29 patients) were T3bN1, 35.48% (11 out of 29) were T3aN0 and 54.83% of the cases (17 out of 29 patients) were T2N0 stage. None of the patients had radiographic metastasis at the time of prostatectomy and the range of Gleason score was between 6 and 9.

**Table 1:** Clinical data for prostate cancer patient cohort

Median Age	65 (49–76)
Median PSA	5.3 (3–27.5)

		***n***	**%**
pTMN Stage	T3N1	1	3.22
	T3N0	11	35.48
	T2N0	17	54.83
Gleason Score	5+4 = 9	1	3.23
	4+5 = 9	4	12.90
	4+4 = 8	1	3.23
	4+3 = 7	4	12.90
	3+4 = 7	16	51.61
	3+3 = 6	3	9.68

**Figure 7:**
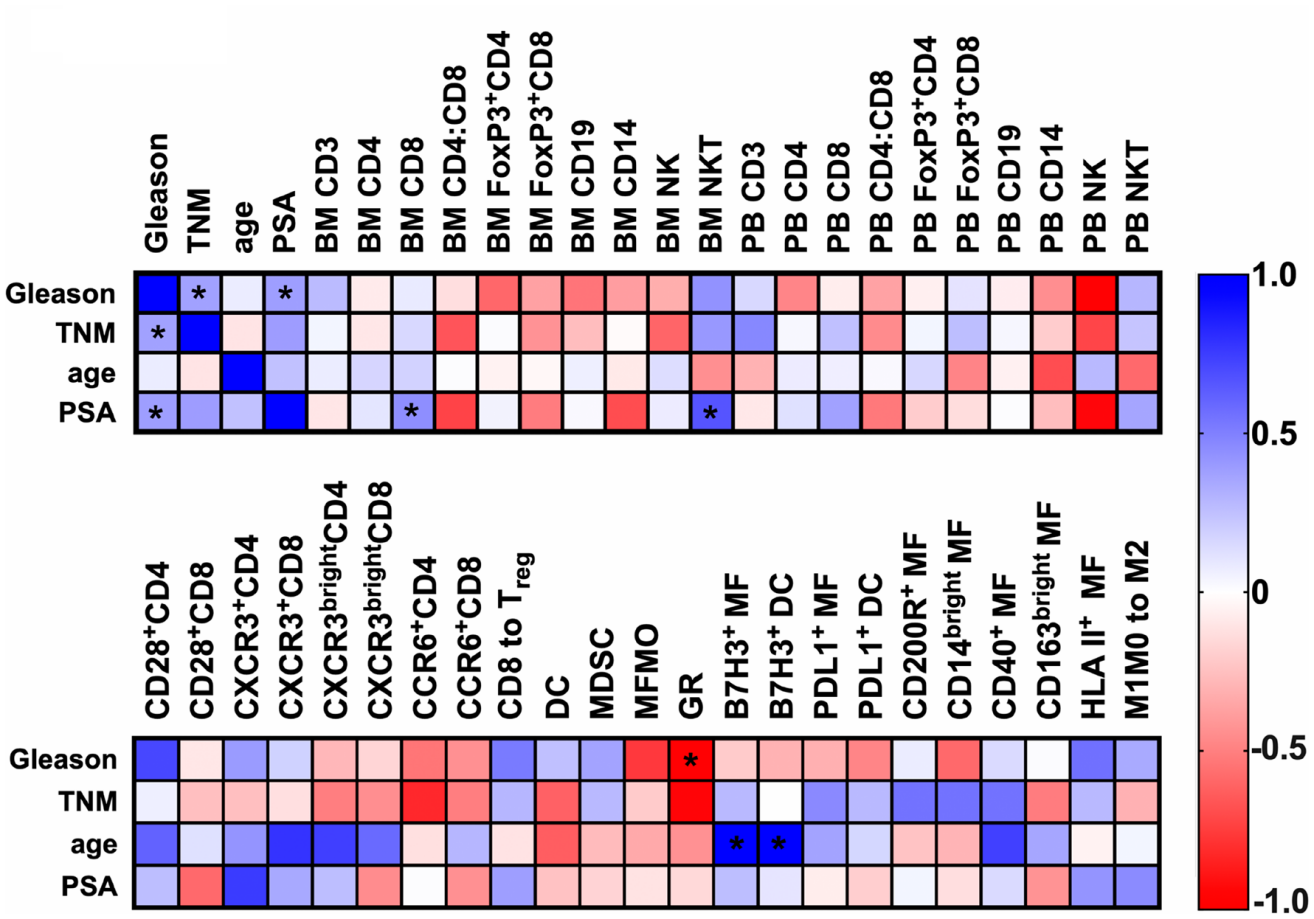
Spearman’s correlation analysis of patient characteristics and immunologic features. Heatmaps represent rho values in Spearman correlation matrices of patient data and immunologic features of bone marrow aspirates and of peripheral blood in PC patients. (top) BM *n* = 11–27, PB *n* = 11–22; (bottom) *n* = 11–14. ^*^ *p* < 0.05, ^**^ *p* < 0.01.

Next, we have conducted multiple correlation analysis between immunologic features and clinical data. Spearman r’ correlations between clinical data and a subset of basic immune features of both BM and PB in the PC cohort are shown in the top matrix of Figure 7. Expectedly, we detected correlations between the Gleason score and TNM stage (represented as T stage in correlation matrix) (Spearman’s rho = 0.37, confidence interval (CI) 0.008468 to 0.6544, *p* = 0.049) and Gleason score and PSA (Spearman rho = 0.38, CI 0.002054 to 0.6604, *p* = 0.043). When analyzing immunologic features, we found a correlation between PSA levels and the frequency of CD8^+^ T cell infiltrates of the total live cellular content of the BM aspirate (Spearman’s rho = 0.42, CI 0.04551 to 0.0.7192, *p* = 0.027). PSA levels at the time of prostatectomy also showed a strong positive correlation with NKT cell enrichment in our patient cohort. This correlation was more enhanced in BM (Spearman’s rho = 0.661, CI 0.08026 to 0.9065, *p* = 0.031) compared to PB (Spearman’s rho = 0.351, CI 0.3338 to 0.7931, *p* = 0.288). Interestingly, NKT yields projected as the frequency of total leukocyte content (CD45^+^ total) also correlated with Gleason score (Spearman’s rho = 0.643, CI 0.0517 to 0.9009, *p* = 0.037), TNM stage (Spearman’s rho = 0.693, CI 0.1390 to 0.9165, *p* = 0.030) beyond PSA levels (Spearman’s rho = 0.615, CI 0.003543 to 0.8918, *p* = 0.049) (Supplementary Figure 3).

The bottom heat map in Figure 7 represents a correlation matrix of a second set of immune features selected from the high granularity assay in BM aspirates. The expression of B7H3 negative costimulatory marker on both DCs (Spearman rho = 0.631, CI 0.03029 to 0.8971, *p* value = 0.041) and MO/MF (Spearman rho = 0.627, CI 0.02267 to 0.8956, *p* = 0.043) subsets also correlated with age. Interestingly, a negative correlation was detected between the yield of GR-like cells within the total CD45^+^ leukocyte content and Gleason scores (Spearman rho = –0.558, CI –0.8452 to –0.02209, *p* = 0.040). A heat map of additional data from the multiple correlation analysis is shown in the Supplementary Material (Supplementary Figure 3).

## DISCUSSION

While the ability of PCs to subvert anti-tumor immunity in the BM has been well documented [25, 26], the immune landscape of bone invasion and the specific pathways that enable tumor cell survival within this immune-rich environment is still poorly understood partly due to the lack of in-depth reference data. In this study, we sought to assess the pre-metastatic immune profile and potential mechanisms of immune evasion permitting development of metastatic foci. Using independently developed companion flow cytometry assays of 21 distinct immune markers, we identified an array of immune alterations within the BM of patients with clinically localized PC. These alterations, which occurred in the absence of detectable bone metastases, may represent key events in the development of bone invasion and suggest that the process of immune subversion in the BM begins prior to the arrival of disseminating tumor cells.

CD4 and CD8 T cells homeostasis is a critical factor, in both primary tumor and at metastatic sites orchestrating anti-tumor activity [27–29]. In our evaluation of the BM lymphoid populations, we identified a significant increase in the CD4/CD8 ratio in PC patients relative to healthy controls while the CD4/CD8 ratio in peripheral blood remained within the healthy reference range [16–18]. Although variations in the immune phenotype have been reported in both blood and BM due to immune senescence, peripheral CD4/CD8 ratios remain largely unchanged over age [16]. In the BM in particular, but in blood and lymph nodes an increase in the CD4/CD8 ratio has been associated with greater risk of BM involvement as well as worse clinical patient outcomes in follicular lymphoma [30]. A decrease of CD4 to CD8 ratio in prostate tissue was reported in a cohort of PC patients following cryo-ablation of tumor nodes reflecting a restoration of T cell homeostasis in response to therapy [31]. Therefore, an increase of BM CD4/CD8 T cell ratio may reflect an important deviation in T cell homeostasis during primary PC pathogenesis, which may ultimately permit bone invasion in these patients. Our current analysis, however, has not identified a specific CD4 or CD8 T cell subpopulation to account for this change. Future studies should explore a potential deviation in T cell differentiation and assess T cell exhaustion and senescence that might also contribute to shifts in CD4-CD8 T cell homeostasis in the pre-metastatic BM niche.

Beside the enrichment of conventional T cell subsets, we also observed an increase of unconventional lymphoid cells including NK and NKT cells in PC BM aspirates. A retrospective study found correlation between peripheral NK cell functionality and time to castration resistance and overall survival in metastatic PC [32]. A high dimensional computational flow analysis of men undergoing routine tests for PC including asymptomatic PC patients with very low PSA (< 20 ng/ml) identified peripheral blood NKT cells as strong biomarker of carcinoma presence in biopsy cores. This study suggested that computational analysis of the peripheral immune profile improved diagnostic accuracy when used in addition to PSA monitoring [33]. Similarly, we found a correlation between PSA levels and BM NKT yields that further supports the hypothesis that peripheral NKT enrichment reflects early PC tumorigenesis. The prognostic value of this biomarker should be further explored in a larger patient cohort with expanded clinical correlates including time to biochemical recurrence.

Myeloid cells, including granulocytes and MDSCs, are also recognized to have major roles in anti-tumor immunity. Although there were no overt differences in the frequency of MDSCs or granulocytes in this study, we did find that the frequency of CD11b^+^ enriched dendritic cells (DCs) was significantly reduced in patients with localized prostate cancer. DCs are the prime antigen presenting cells involved in T cell activation and tumor immune surveillance [34]. While they can initiate dramatic immune-mediated tumor rejection, the immunogenic activity of DCs is directly linked to their abundance within the tumor microenvironment. Preclinical data in a murine model of BM metastasis demonstrated PC-mediated inhibition of DC generation [35]. Thus, the reduction of DC number in patients with localized PC suggests that the DC population at this early stage of disease may be less effective at initiating an adaptive immune response against tumor cells within the BM.

In the monocyte/macrophage populations, we identified an increase in expression of the immune checkpoint molecule, B7H3. B7H3 is a regulator of T cell function [36], and is related to both AR-signaling and the immune reactome. B7H3 showed correlation with Gleason score, cancer stage and poor oncologic outcomes in a large cohort of prostatectomy specimens [37]. The role of the B7H3 immune checkpoint in regulation of the pre-metastatic PC immune microenvironment should be further investigated. A Phase II clinical trial is currently evaluating the anti-tumor effect and immunogenicity of anti-B7H3 (Enoblituzumab) neoadjuvant therapy given to patients prior to radical prostatectomy (NCT02923180).

M2-like tumor-associated macrophages have been reported in both primary prostate carcinomas and in castrate-resistant PC [38] and may have potential as a biomarker for biochemical recurrence in PC [39]. In current study, we found no evidence of a macrophage polarization shift between healthy donors and PC patients. However, our scope has been limited to a binary phenotypic characterization in a relatively small cohort and functional assessment of the BM MOMF should be further explored in PC BM.

Our observations suggest that an active interplay between myeloid and lymphoid compartments is present in BM in the absence of detectable metastasis, and raises the hypothesis that an immunosuppressive BM environment predisposes patients to recurrent disease. Therefore, immune aberrations could potentially be detected prior to disseminated tumor cells (DTCs) and radiographic evidence of bone metastases.

BM samples of prostatectomy patients have been previously assessed with various rare-event detection methods in an attempt to aid patient stratification. A recent study by Chalfin et al. which included 12 out of 27 BM from the current samples concluded that the detection of DTCs in BM was a rare occurrence [40]. Herein, we show that aberrations in BM immune homeostasis are more common events than DTCs, suggesting that detection of immune alterations could potentially be a more useful stratification marker to predict patients at higher risk for recurrence. While the sample size and duration of follow-up in this study were not sufficient to make definitive conclusions, the array of lymphoid and myeloid alterations in patients with organ-confined PC are consistent with an increased risk for BM metastases. Future studies evaluating the multi-cellular BM microenvironment in both aspirates and in biopsies and clinical follow-up to identify patients with early biochemical recurrence are critically needed to understand whether immune suppressive events precede early BM invasion and if immune alterations in patients with PC could be therapeutically targeted to prevent bone metastasis.

In this paper, we describe a comprehensive interrogation of the immune compartment in BM from patients with clinically localized PC. Our findings demonstrate that immunosuppressive alterations of the lymphoid and myeloid compartments within the BM can occur early in PC pathogenesis. In the process of tumor cell metastasis, such alterations could promote the outgrowth of disseminating prostate cancer cells within the BM. As such, the presence of these alterations may hold important prognostic significance as well as predict which patients might benefit from more aggressive therapy. Our report provides the first in-depth analysis of the BM immune phenotype in primary, localized PC. These findings highlight the importance of the unique niche present in bone and bone marrow, and provide rationale for further research of the osseous immune microenvironment in patients with prostate cancer.

## MATERIALS AND METHODS

### Patients

The study was approved by the institutional review board at University of Wisconsin and Johns Hopkins University. The study enrolled a total of 29 prostate cancer patients providing written consent to collect BM aspirates and peripheral blood while undergoing radical prostatectomy for presumed localized disease. From the 29 donors, we have received and analyzed a total of 27 BM aspirates and 24 PB samples, of which 22 samples were completely matched. Patient characteristics including Gleason score, TNM stage, age, PSA, pertinent laboratory values, and clinical outcomes are summarized in Table 1. Healthy BM aspirates were obtained from 5 male BM donors at Johns Hopkins University, and from left over BM filters of 5 healthy individuals [41] providing BM for donor transplantation at the University of Wisconsin BM Transplant program IRB# 2016-0298.

### BM and peripheral blood processing

Blood was collected into K_2_EDTA Vacutainer tubes (BD Biosciences, USA). BM aspirates were collected in a heparinized tube and inverted immediately followed by an hour digestion with Heparin (100 IU/ml) and DNase I (100 IU/ml). Mononuclear cells were isolated from pre-processed BM samples using density gradient centrifugation overlaid on LSM media (Corning, USA) following manufacturer’s protocol. PBMCs from peripheral blood (PB) were isolated on Ficoll-Paque (GE, USA) gradient.

### Flow cytometry

Using multicolor flow cytometry, the immune compartment in matched blood and BM samples were examined. ~2 million PBMCs were stained with a comprehensive panel for immune staining. The initial cohort of 15 patient samples were analyzed with a basic panel utilizing Ghost Dye™ Violet 510 (Tonbo Biosciences, San Diego, CA, USA), human Fc blocker (BD Biosciences, San Jose, CA, USA) and antibodies including CD45-PE, CD19-FITC, CD4 Brilliant Violet™ 421, CD8 APC-Cy7, FoxP3-APC and CD14 Brilliant Violet™ 786. To gain higher granularity data on activation and functional polarization the assays were expanded into two panels including CD45, CD19, CD14, CD3, CD11b, CD56, CD163, CD200R, HLA II, B7H3, PDL1, CD40 in Panel I and CD45, CD4, CD8, FoxP3, CD82, CXCR3, CXCR5, CCR4, CCR6, CD103 in Panel II, with further details summarized in Supplementary Table 1. Cells were stained with human Fc blocker, Ghost Dye™ Violet 510 stain and fluorescently conjugated antibodies for immune labeling of surface markers. Surface staining was followed by a washing step and fixation with 2% PFA (Cytofix, BD Biosciences, San Jose, CA, USA) in Panel I or fixation & permeabilization for intracellular staining with the FoxP3 Staining kit following the manufacturer’s protocol (eBioscience portfolio by Thermo Fisher Scientific, Waltham, MA, USA) in Panel II. Samples were acquired following pre-acquisition instrument standardization with Mid-Range Ultra Rainbow Fluorescent Particles (Spherotech, Lake Forest, IL, USA) on a BD LSR II instrument at the UWCCC Flow Cytometry Laboratory. Gating controls included Internal Negative Controls (INCs) and Fluorescent Minus One (FMO) controls [42]. Due to limited sample size, the FMOs were established on an independent cohort of donor PBMC with matched assay and instrument settings to confirm no spectral spillover into positive gating areas. Representative plots for gating strategies are included in Figures 1A, 5A and Supplementary Figure 2. Monocytes (CD45^int^SSC_int_) served as INC to validate gating thresholds for CD28, CXCR5, CXCR3 and CD103 expression [43, 44]. The CD45^-^SSC_low_ subset served as INC for CCR4 and FoxP3 expression. CCR6 expression was cross-examined on CD4^+^CD8^+^ double positive lymphocytes and the CXCR3^bright^ subset to confirm gating thresholds [45, 46]. Lymphocytes (CD45^bright^SSC_low_) served to confirm baseline thresholds for myeloid markers (Supplementary Figure 2B). B7H3 and PDL1 are primarily expressed in myeloid leukocytes while the majority of lymphocytes do not express these proteins and align with FMO baselines [47–49]. The CD3^+^ fraction and the majority of lymphocytes are both negative for CD40 and CD19^+^ cells serve as an internal positive control [50]. CD19^+^ B cells served to set baseline threshold for CD200R expression [24]. CD163 is expressed by macrophages and is upregulated on M2 macrophages [22]. For the M2 analysis, we gated on the CD163^bright^ subset [51]. The CD163^bright^, CD14^bright^ and HLA II^bright^ subsets were gated on the top, brightly expressing, spectrally distinct subsets within those positive fractions.

The flow cytometry data was analyzed by FlowJo Ver. 9.9.6 (BD Biosciences, San Jose, CA, USA).

### Statistical analysis

Statistical comparison between groups was made with Welch’s *t*-test in Prism 8 Version 8.4.2 (GraphPad Software, San Diego, CA, USA). Correlation analysis of patient data and immune phenotype were done with Spearman’s r in Prism 8.

## SUPPLEMENTARY MATERIALS



## Abbreviations

BM: bone marrow
GR: granulocyte
DC: dendritic cell
FSC: forward scatter
MDSC: myeloid-derived suppressor cell
MO/MF: monocyte/macrophage
NK: natural killer cell
NKT: natural killer T cell
PB: peripheral blood
PC: prostate cancer
PSA: prostate-specific antigen
SSC: side scatter
TNM: tumor node metastasis staging
