# Supplementary material for: Immune profiling of the bone marrow microenvironment in patients with high-risk localized prostate cancer
Source: Oncotarget. 2020 Nov 17;11(46):4253–65. doi: 10.18632/oncotarget.27817 (PMC7679037; doi:10.18632/oncotarget.27817)
Supplement: Supplementary file 1 [file oncotarget-11-4253-s001.pdf]

Immune profiling of the bone marrow microenvironment in patients with high-risk localized prostate cancer

SUPPLEMENTARY MATERIALS

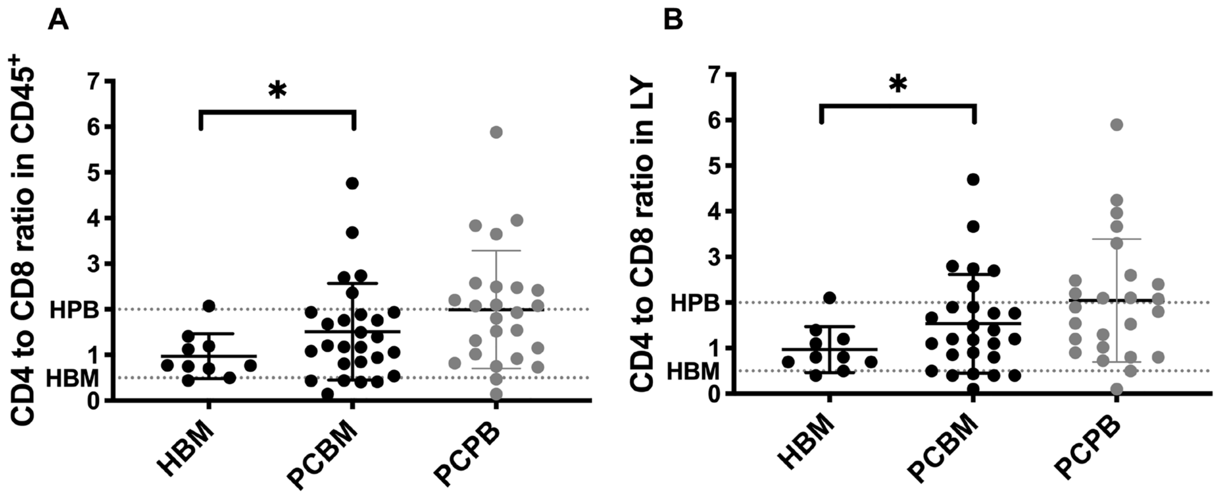

Supplementary Figure 1: CD4 to CD8 ratio calculated as frequency of CD4$^+$ over frequency of CD8$^+$ cells in the (**A**) CD45$^+$ infiltrate or (**B**) in the CD45^{bright}/SSC$_{low}$ lymphocyte subset. Healthy bone marrow (HBM), PC bone marrow, PC blood (PCPB). HBM $n = 10$, PCBM $n = 27$, PCPB $n = 24$. Data expressed as Mean +/–SD, $p < 0.05$.

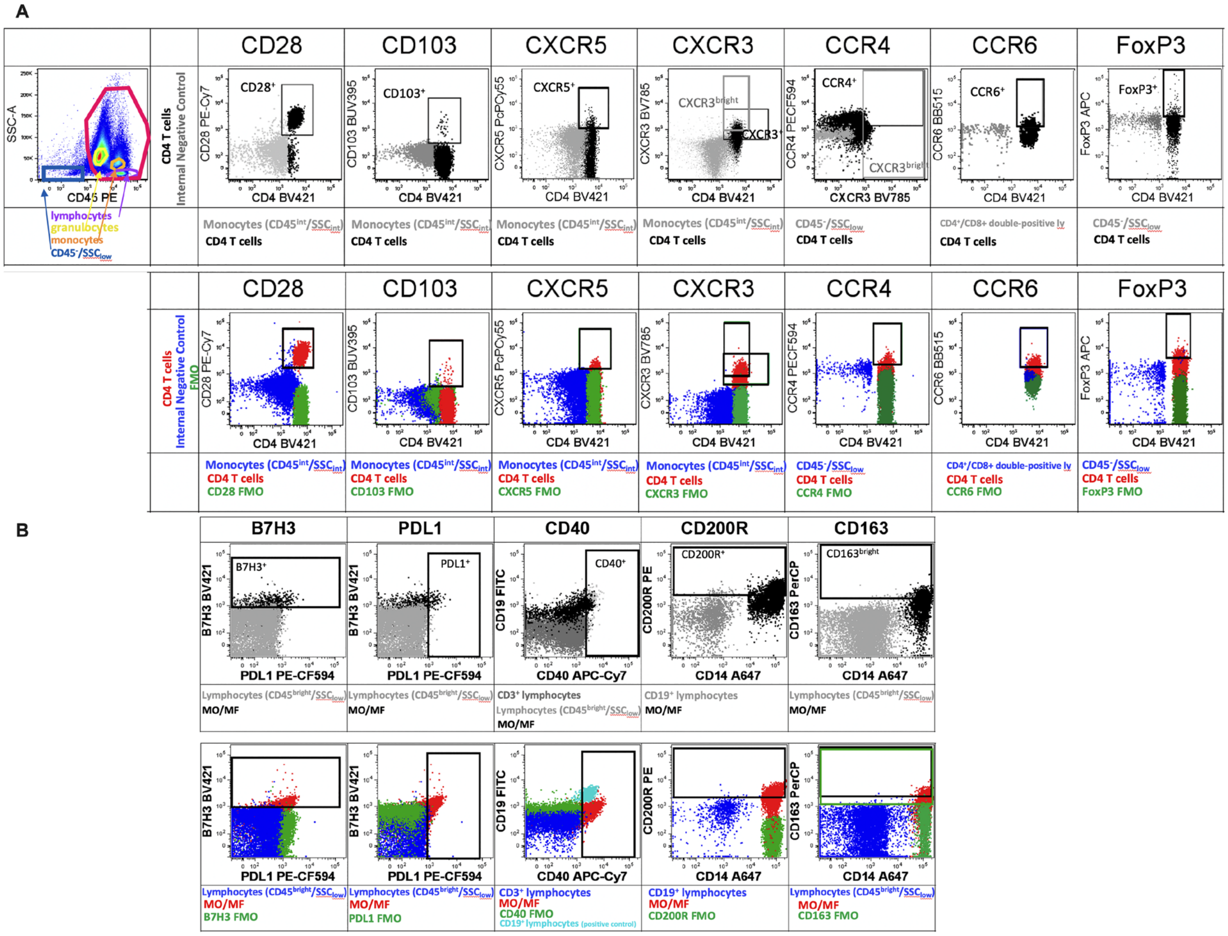

Supplementary Figure 2: Representative gating controls for T cell and myeloid cell analysis. (**A**) In the upper row, representative dot plots show gating thresholds for T cell analysis with an overlay of internal negative control (INC) populations (in grey) on the representative CD4 T cell subset (in black). The second row shows FMO controls (full stain on $CD4^{+}$ in red, full stain INC subset in blue, FMO stain on $CD4^{+}$ in green). Monocytes (CD45+SSCint; shown in grey on overlay; circled orange on CD45 vs SSC Panel II dot plot) served as INC to validate gating thresholds for CD28, CD103, CXCR5, and CXCR3 expression. The $CXCR3^{bright}$ events spectrally segregated within the CXCR3+ subset and these cells preferentially expressed CXCR3 and not CCR4. The CCR4 gate was further validated on the $CD45^{-}SSC_{low}$ subset (outlined blue on dot plot). The $CD45^{-}SSC_{low}$ subset also served as INC to confirm thresholds for FoxP3 and CXCR5 (not shown). The CD4+CD8+ double positive lymphocyte subset and CXCR3bright cells (not shown) were used as gating control for CCR6 expression. (**B**) Representative dot plots show myeloid gating strategies on the MO/MF subset with INCs (top row; MO/MF in black, INC in grey) and FMOs (second row; full stain MO/MF in red, full stain INC in blue, FMO on MO/MF in green). Lymphocytes ($CD45^{bright}SSC_{low}$; circled in purple on (A) top left) served to set baseline thresholds for myeloid markers. CD3+ T cells and the majority of lymphocytes used as INC for the CD40 analysis with the $CD19^{+}$ subset shown (in light blue) as positive control. CD19+ B cells served an INC to set threshold for CD200R expression. CD163 is expressed by macrophages and is upregulated on alternative macrophages ($CD163^{bright}$). We gated on the $CD163^{bright}$ subset that spectrally segregated off the main population. Lymphocytes show $CD163^{-}$ expression that aligns with FMO.

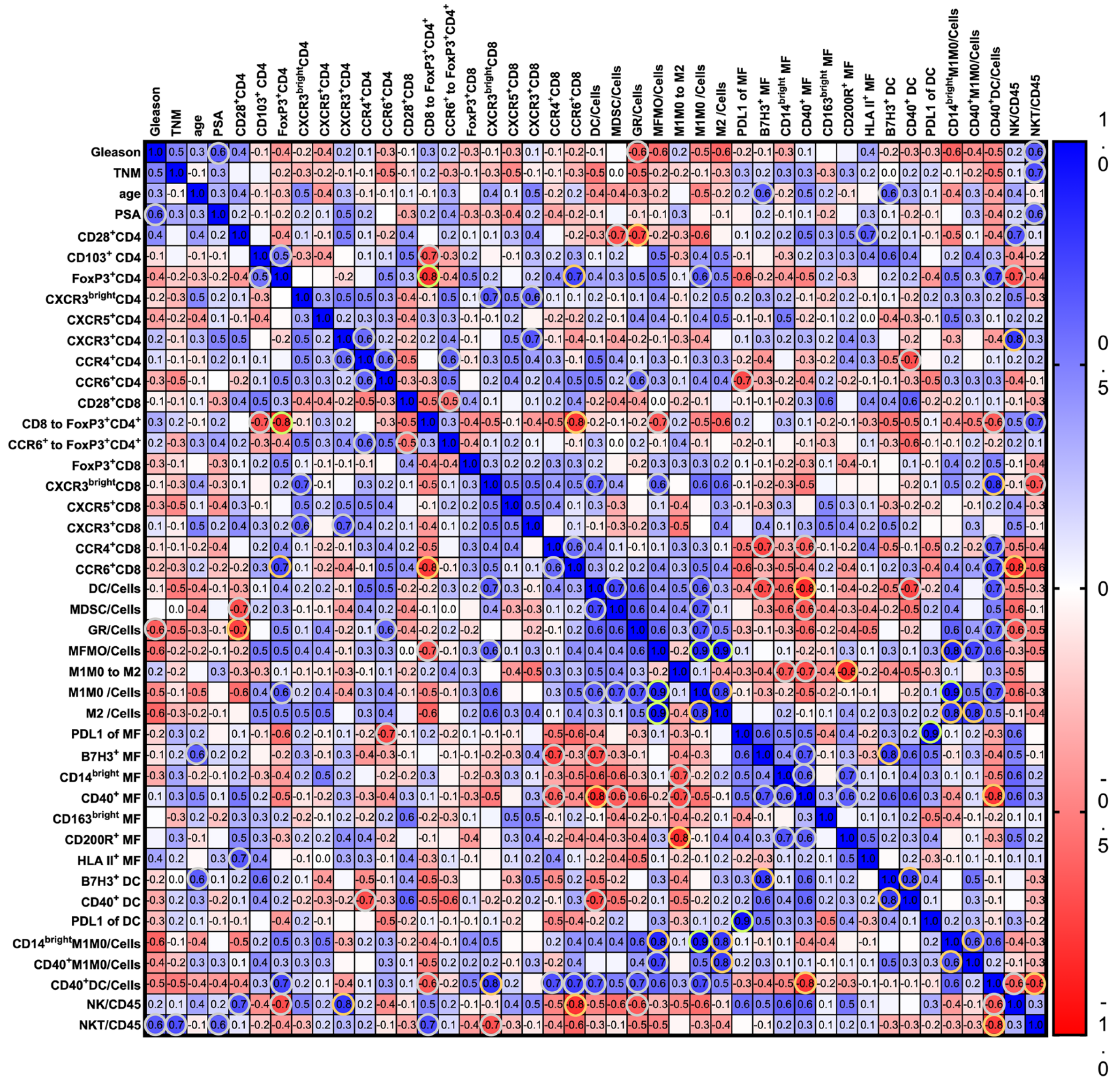

Supplementary Figure 3: Spearman's multiple correlation analysis between features of the bone marrow immunologic milieu and clinical data. Heatmap represents Spearman's Rho values. Blank cells have values outside defined range. /Cells = frequency of total live cells; /CD45 = frequency of total $CD45^+$; $CD56^+$ cells gated directly from $CD45^+$. n = 11 to14. Grey circle represents $P < 0.05$, orange circle $p < 0.01$ and green circle $p < 0.001$.

Supplementary Table 1: Multi-color flow cytometry assay panels designed to interrogate myeloid and lymphoid immune subsets of BM aspirates

**Panel I**		
**marker**	**fluorochrome**	**purpose**
Live/Dead fixable	Ghost Dye™ 510	Dead exclusion; reduce autofluorescence
CD45	redFluor™ 710	leukocyte common antigen
CD19	FITC	B cell marker
CD3	PE-Cy7	T cell marker
CD11b	Brilliant Violet™ 786	myeloid panmarker
CD14	Alexa™ 647	monocyte-associated differentiation
CD56	Brilliant UltraViolet™ 737	natural killer cell marker
CD40	APC-Cy7	positive co-stimulation
PDL1	PE-CF594	negative co-stimulation
B7H3	Brilliant Violet™ 421	negative co-stimulation
CD200R	PE	M2 macrophage associated marker
CD163	PerCP	M2 macrophage associated marker
HLA-DP/DQ/DR	Brilliant UltraViolet™ 395	activation and differentiation marker
Fc Block™	n/a	reduce aspecific binding

**Panel II**		
**marker**	**fluorochrome**	**purpose**
Live/Dead fixable	Ghost Dye™ 510	Dead exclusion; reduce autofluorescence
CD45	PE	leukocyte common antigen
CD28	PE-Cy7	T cell activation marker
CD4	Brilliant Violet™ 421	CD4 T cell
CD8	Brilliant UltraViolet™ 805	CD8 T cell
FoxP3	APC	regulatory T cell marker
CXCR5	PerCP-Cy5.5	Tfh/Tfc follicular T cell marker
CCR6	Brilliant Blue™ 515	Th17/ Tc17 marker
CXCR3	Brilliant Violet™ 786	Th1/ Tc1 marker
CCR4	PE-CF594	Th2/Tc2marker
CD103	Brilliant UltraViolet™ 395	TIL marker
Fc Block™	n/a	reduce aspecific binding
